# Drifting

**Published:** 1861-10

**Authors:** 


### DRIFTING.

"Revolutions never go backward," said some renowned name. Multitudes have repeated the sentiment since, and it has almost become an axiom. It is a great untruth. Revolutions often have a reactive power, and the people are left in a worse condition than before they revolved; they turned up, and then they turned down. "Reform" is another name for revolution, more modest, less startling; meaning to intimate that the foundation will be left, first principles will remain undisturbed, but will be modified in their application. Once upon a time, Presbytery got up a revolution, and bestrode the high horse of reform as to Episcopacy and Papacy, and so sturdy were they in their work, so thorough, they made such "a clean sweep," as to matters and things in general, that they reformed some things which had, perhaps, have been better let alone. This is not a theological article—it relates specially to practical life, as will be presently seen. One of the things against which the battering-rams theologic were particularly directed, was the abolition of fast days, and feast days, and saints' days; they left not a single day standing—not even "old father Christmas" day. They won't even have a sermon on that day, unless it should happen to come on Sunday. The fact is, the sober Presbyterian has no day at all, from Genesis to December. Many of them won't allow their children to set off squibs or fire-crackers on the Fourth of July. They are so sober, so sedate, Presbyterians are, they seldom crack a smile on the street or highways. No doubt they at times let off the bottled mirth, else consequences might be disastrous. In fact, we remember a "case," as doctors say.

There is a seat in a church we know of, which is almost never vacant, occupied by a sober Protestant; his eye is fixed on the minister, from beginning to end—we do not remember ever to have seen it winked; the chapter and verse are always hunted out, and the psalms sung with an unction, especially the doxology. So we gradually gave to the gentleman our most respectful consideration. Later on in the history of things, we happened to be one of about a hundred men, brought together by design. As far as we could judge, they all seemed to be eminent men in their line; there were generals, and governors, and commodores, and millionaires, and, with the exception of a doctor or two, there was nothing less than a senator present. There were Astor, and Scott, and Seward, and Brooks, and Greeley, and Morgan, and Vanderbilt, and Cooper, and Cyrus W., &c. When the oysters came, and the champagne and the brandy, about eleven o'clock, there was exceeding joy. We watched. There was one gentleman who took the lead. Didn't he tip and twirl his hat to the loud hip, hip, hurrah, at Seward's speech, and louder still at the call for "Brooks;" and what hearty mirth at the exhaustion of each goblet! We had always heard that the most mirthful people before folks, were the most solemncholy when alone, and here was a "case" at hand, for we recognized the lineaments of the dutiful hearer before alluded to. And now we come to the point: Protestants reformed all feast days and saints' days out of the calendar. But is not the revolution going backward with a vengeance? Instead of remembering the birthday of persons remarkable for their piety, with reverence for their virtues, with humility for our shortcomings of them, and with penitential resolves to be more like what they were reputed to be—we repeat it—instead of saints' days for fastings, we have sinners' nights for feastings. Tom Paine's birthday is celebrated by feasts and liquor-drinking, by descendants of the Pilgrim Fathers. And, later still, over this whole land, men of all creeds and parties have laid aside their differences; men of high social position, men who have wives and daughters and sons, lawyers and *literateurs*, all tip the glass to the same sentiments, and hurrah at the same speeches, and eat and drink and carouse until the small hours of the morning; and in memory of what?—that, a hundred years earlier, was born a drunkard and a debauchee, a man who wasted his great powers of poetry, of wit, of sarcasm—worse than wasted them; he prostituted them, as far as lay in his power, to purposes of ridicule against the religion of his fathers, and of his country. A blasphemous man was he; and whether his hypocrisy, his inconsistencies, or his maudlin sentimentalities were greatest, we cannot say. A man abuses himself with liquor and license, and then turns round and abuses the world for not taking him in its arms, and providing for all his wants. If he had been treated with less attention, he would have been less a drunkard. The poet of Ayr and of Bonny Doon was great in sentiment. There is a sweetness in his lines above all others ever written. As to these same sentiments, and for their sweet things, let an honorable and respectful remembrance be had by all who have been thrilled with his poesy. But when it comes to a semi-deification of the virtues of a man who had no virtue at all, simply because he made delightful poetry, that is going rather far; it is reform run mad; it is revolution gone to seed; it is the glorification of the profane, with idolatry and wine, instead of the remembrance of the good, with profitable humiliations.

When it comes to this, that, after a dinner and a dram, eminent men get up, not in the light of day, but in the glare of gas, and apologize for beastly drunkenness, and for the debauchery of women, as the overflowing hilarity of a genial soul, the outbreakings of an affectionate nature—bah! At the bacchanal of Burns, in Boston, great men asserted that drunkenness and licentiousness, after the manner of their hero, were "symbols of the highest type of manhood;" that "men who controlled their passions and led virtuous lives, might win respect, but could not excite love." Such language as this, at the homestead of the Puritans, where wealth, and refinement, and cultivation, and high scholarship, make their home, may well "cause us pause," and inquire whither are we drifting?

